# Rotavirus-Mediated Prostaglandin E_2_ Production in MA104 Cells Promotes Virus Attachment and Internalisation, Resulting in an Increased Viral Load

**DOI:** 10.3389/fphys.2022.805565

**Published:** 2022-01-28

**Authors:** Willem J. Sander, Gabré Kemp, Arnold Hugo, Carolina H. Pohl, Hester G. O’Neill

**Affiliations:** ^1^Department of Microbiology and Biochemistry, University of the Free State, Bloemfontein, South Africa; ^2^Department of Animal Science, University of the Free State, Bloemfontein, South Africa

**Keywords:** rotavirus, viroplasm, prostaglandin E_2_, fatty acid supplementation, internalisation, attachment, lipid droplets

## Abstract

Rotaviruses are one of the leading causes of severe dehydrating diarrhoea in infants and children under the age of five. Despite the introduction of vaccines, disease burden remains high in sub-Saharan Africa, with no known anti-viral treatments available. During early infection rotavirus attaches to several cellular receptors and enters the cells by either clathrin-dependent or -independent endocytosis. Prostaglandin E_2,_ an abundant eicosanoid, is produced from arachidonic acid during rotavirus infection and inhibition of prostaglandin E_2_ formation have a deleterious effect on rotavirus infection. In this study, MA104 cells were supplemented with γ-linolenic acid (GLA), a precursor of arachidonic acid. Infection of supplemented cells with rotavirus SA11 led to a depletion in the relative percentages of GLA and arachidonic acid which coincided with an increased production of prostaglandin E_2_ as monitored by ELISA. Confocal microscopy demonstrated that prostaglandin E_2_ co-localises with the viroplasm-forming proteins, NSP5 and NSP2. Due to the known association of viroplasms with lipid droplets and the fact that lipid droplets are sites for prostaglandin E_2_ production, our results indicate a possible role for viroplasms in the production of rotavirus-induced prostaglandin E_2_. Replication kinetics showed that inhibitors, targeting the biosynthesis of prostaglandin E_2_, had negative effects on rotavirus yield, especially during the early stages of infection. Using flow cytometry and prostaglandin E_2_ addback experiments, we show that prostaglandin E_2_ enhances the attachment and internalisation of rotavirus in MA104 cells indicating a possible role for prostaglandin E_2_ during clathrin-mediated rotavirus entry. The production of prostaglandin E_2_ during rotavirus infection could serve as a possible target for anti-viral treatment.

## Introduction

Rotavirus (RV), a member of the *Reoviridae* family, causes severe dehydrating diarrhoea in infants and young children (Estes and Greenberg, 2013). Despite the global introduction of several RV vaccines, mortality in Sub-Saharan Africa remains high (Godfrey et al., 2020). The 11-segmented dsRNA genome of RV encodes six structural viral proteins (VP) and five or six non-structural proteins (NSP; Estes and Greenberg, 2013). Although RV attachment and internalisation are not well understood it is known that RV binds to several cell receptors, where after internalisation occurs rapidly by either clathrin-dependent or -independent endocytosis, depending on viral strain (Arias and López, 2021). During viral replication, RV forms cytoplasmic inclusion bodies, termed viroplasms, which are required for assortment, genome replication and formation of progeny viral particles (Papa et al., 2021). The formation of viroplasms requires host lipid droplets (LDs) and several viral proteins, specifically NSP2 and NSP5 (Cheung et al., 2010; Kim et al., 2012; Gaunt et al., 2013; Crawford and Desselberger, 2016).

Lipid droplets are organelles that play diverse roles in the physiology and pathophysiology of cells (Walther et al., 2017; Brink et al., 2021). The major components, making up the neutral core of LDs, are triacylglycerols and sterol esters, which serve as energy stores during nutrient deprivation (Jackson et al., 2016). This core is surrounded by a phospholipid monolayer that contains diverse membrane-bound proteins (Bartz et al., 2007). Some of these proteins, such as viperin, immunity-related GTPases, lipoxygenases and cyclooxygenases (COX), are involved in the production of lipid mediators, which play crucial roles during the immune response to viral infections (Accioly et al., 2008; Bozza et al., 2011; Monson et al., 2021). Important examples of such lipid mediators are the eicosanoids belonging to the prostaglandins, which modulate inflammation (Ricciotti and FitzGerald, 2011).

Prostaglandins are bioactive molecules that are derived from arachidonic acid (AA; Phipps et al., 1991; Ricciotti and FitzGerald, 2011). The most common PGs, prostaglandin E_2_ (PGE_2_), are produced by all mammalian cell types and regulate several physiological processes, including blood pressure, fertility, gastrointestinal integrity, immunity and inflammation (Ricciotti and FitzGerald, 2011). During viral infections, the production of PGE_2_ can, to the detriment of the host, be modulated to benefit the virus (Sander et al., 2017). Rotavirus has been shown to increase PGE_2_ levels *in vitro* during infection of human colorectal adenocarcinoma (Caco-2) cells (Rossen et al., 2004) and *in vivo* in piglets (Zijlstra et al., 1999). Increased levels of PGE_2_ were also detected in the stool of RV-infected children and treatment with the COX inhibitor, acetylsalicylic acid, reduced the duration of diarrhoea (Yamashiro et al., 1989). In addition, Hagbom et al. (2012) suggested that the ability of PGE_2_ to stimulate the excretion of water could contribute to RV disease progression. A previous study suggested a role for PGE_2_ during the early stages of infection, with no effect on viral RNA, while decreases in protein synthesis and production of viral progeny were observed (Rossen et al., 2004).

To further elucidate the role of PGE_2_ during RV infection, MA104 cells supplemented with the AA precursor, γ-linolenic acid (GLA), were infected with RV and compared to unsupplemented infected and uninfected cells. We show that RV decreases the relative percentage of AA (a precursor to PGE_2_) and increases PGE_2_ levels. The increase in PGE_2_ coincides with an increase in viral replication in a time- and dose-dependent manner. Most significantly, we show that the inhibition of PGE_2_ biosynthesis affects RV replication at the attachment and internalisation stage.

## Materials and Methods

### Cells, Virus and Inhibitors

The African Green Monkey kidney (MA104) cell line was maintained in Dulbecco’s modified Eagle medium (DMEM; Gibco), supplemented with 5% (v/v) foetal bovine serum (FBS; Gibco), 1% (v/v) Penicillin-Streptomycin-Amphotericin B Mixture (10,000 U, 10,000 μg and 25 μg/ml; Lonza) and 1% (v/v) nonessential amino acids (NEAA; Lonza) at 37°C and 5% CO_2_. Subconfluent cells were supplemented with 50 μM GLA (Thermo Fischer Scientific) for 24 h according to Tanaka et al. (2001). Rotavirus simian agent 11 strain (SA11; Mlera et al., 2013) was used to infect MA104 cells and viral yield was determined using 50% tissue culture infectious doses (TCID_50_; Reed and Muench, 1938). To facilitate infection, pancreatic porcine trypsin type IX (1 μg/ml; Sigma-Aldrich) was added during all viral replication experiments.

The non-specific cyclooxygenases inhibitor, indomethacin; the COX-1-specific inhibitor, SC-560; the COX-2-specific inhibitor, Celecoxib; cytoplasmic phospholipase A_2_ inhibitor, CAY10502 and PGE_2_ were obtained from Sigma-Aldrich and resuspended in 100% (v/v) dimethyl sulfoxide (DMSO; Sigma-Aldrich). Cellular toxicity for the inhibitors and DMSO was evaluated with the XTT assay (Sigma-Aldrich; Supplementary Figure S1). Final DMSO concentration did not exceed 1%.

### Cellular Lipid Analysis

MA014 cells (4.9 × 10^6^ cells/ml) were seeded into 175-cm^3^ flasks (Thermo Fischer Scientific) and allowed to grow to 80% confluence. After 24 h incubation with supplemented GLA at 37°C, the MA104 cells were infected with SA11 [multiplicity of infection (MOI) = 1] for 16 h and rinsed three times with Phosphate-buffered saline (PBS) before they were scraped from the plates. Mock controls, which were not infected, were processed in parallel. Wet biomass was determined and total lipids extracted with chloroform:methanol (2:1; Folch et al., 1957). The total lipids were separated into neutral and phospholipid fractions with 500 mg aminopropyl silica cartridges (Varian) according to the method of Bossio and Scow (1998). The eluates were collected and dried under N_2_ at room temperature. Fatty acid methyl esters of the lipid fractions were prepared using methanol–BF_3_ and quantified using a Varian 430 gas chromatograph, with a fused silica capillary column (Chrompack CPSIL 88, 100 m length, 0.25 mm ID, 0.2 μM film thickness). The column temperature was 40–230°C (hold 2 min; 4°C/min; hold 10 min). Fatty acid methyl esters in hexane (1 μl) were injected into the column using a Varian 8400 Autosampler with a split ratio of 100:1. The injection port and detector temperatures were maintained at 250°C. Hydrogen, at 45 psi, functioned as the carrier gas, while nitrogen was employed as the makeup gas. Galaxy Chromatography Data System software recorded the chromatograms. Fatty acid methyl esters were identified by comparing the relative retention times of peaks from samples with those of standards. Fatty acids were expressed as the percentage of each individual fatty acid relative to the total of all fatty acids present in the sample.

### Prostaglandin E_2_ Quantification and Authentication

MA014 cells (0.3 × 10^6^ cells/ml) were seeded into 6-well plates (Thermo Fischer Scientific) and allowed to grow to 100% confluence. The amount of secreted PGE_2_ in the supernatants of mock- or RV-infected (MOI = 5), both in the presence and absence of inhibitors, was determined at 2 and 4 h post-infection, using an ELISA (Cayman Chemicals) according to the manufacturer’s protocol. In order to determine the effect of viral load on PGE_2_ production, PGE_2_ was measured at different MOIs (0.1; 1; 10). LC-MS/MS was used for authentication of PGE_2_ production. Briefly, the supernatant of each infection was divided into two 500 μl aliquots. One of the 500 μl aliquots was spiked with PGE_2_ to a final concentration of 100 ng/ml. In order to prevent free radical catalysed lipid peroxidation, citric acid (Sigma-Aldrich) and butylated hydroxytoluene (Sigma-Aldrich, United States) were added to a final concentration of 80 mm and 0.1% (v/v), respectively. Prostaglandins were extracted by addition of 2 ml hexane/ethyl acetate (1:1, v/v), followed by vortex mixing for 1 min. After centrifugation for 5 min at 1,000 × *g* at 4°C, the upper, organic phase was removed (Caoa et al., 2008). The extraction procedure was repeated, and the organic phases were combined and evaporated to dryness under a stream of nitrogen gas. Twenty microliters of each sample were separated on a C18 column (Luna 3 μM C18, 150 × 3 mm, Phenomenex) at a flow rate of 200 μl/min, using 0.1% (v/v) formic acid (mobile phase A) and acetonitrile with 0.1% formic acid (mobile phase B). The column was equilibrated and loaded at 20% of mobile phase B, increasing to 42.5% B over 50 min, 95% B for 10 min, followed by re-equilibration at 20% B for a total run time of 70 min. Ion spray voltage was set at 4,500 V, nebuliser gas (GS1) was at 40 psi and heater gas (GS2) at 30 psi with the heater temperature set at 400°C. Samples were analysed using a 4000 QTRAP hybrid triple quadrupole ion trap mass spectrometer (AB Sciex) and Shimadzu UFLC stack with LC-20AB binary pump and SIL-20A HT Autosampler as front end. All data acquisition and processing were performed using Analyst 1.5.2 (AB SCIEX) software. To analyse the sample, a targeted Multiple Reaction Monitoring (MRM) workflow was followed on the instrument. All compound- and source-dependent parameters were optimised using compound optimization in Analyst 1.5.2. The targeted analysis for the extracted PGE_2_ used 5 MRM transitions: 351.17 > 315.2; 351.17 > 271.2; 351.17 > 333.3; 351.17 > 189.0; and 351.17 > 235.1. Only if all five transitions were recorded at the same retention time would the presence of PGE_2_ be confirmed.

### Confocal-Laser Scanning Microscopy

To determine the co-localization between viroplasms and PGE_2_, MA104 cells (0.1 × 10^6^ cells/ml) were seeded into coverslips in 24-well plates (Thermo Fischer Scientific) and allowed to grow to 80% confluence before being infected at a MOI of 5. The cells were then processed according to Bandeira-Melo et al. (2011) with slight modifications, 2 and 4 h post-infection. Briefly, MA104 cells were fixed and permeabilised with a mixture of 0.5% (w/v) 1-ethyl-3-(3-dimethylamino-propyl) carbodiimide hydrochloride (Sigma-Aldrich) and 1% (v/v) paraformaldehyde in PBS, for 1 h at 37°C. In addition to cross-linking the carboxyl groups of eicosanoids to the amines of adjacent proteins localised at eicosanoid-synthesizing sites, 1-ethyl-3-(3-dimethylamino-propyl) carbodiimide hydrochloride is also able to both fix and permeabilise cells (Bandeira-Melo et al., 2011). After fixation and permeabilisation, the cells were blocked with 1% (w/v) bovine serum albumin (BSA) plus 0.1% (v/v) Triton X-100 in PBS and washed with PBS before overnight incubation (4°C) with PBS containing 1% BSA and 1:100 anti-PGE_2_ monoclonal antibody (Cayman Chemicals). The cells were washed three times and then incubated in PBS containing 1% BSA and 1:500 rabbit anti-NSP2 (kind gift from Prof. AC Potgieter, Deltamune, South Africa) and/or rabbit anti-NSP5 peptide (KSPEDIGPSDSASNC; GenScript) for 1 h at 37°C. The cells were then washed three times and incubated with 1:500 anti-mouse Alexa-Flour 488 (Thermo Fischer Scientific) and 1:500 anti-rabbit Alexa-Flour 647 (Thermo Fischer Scientific) for 1 h at 37°C. Cells were extensively washed and incubated with 300 nM DAPI (Thermo Fischer Scientific) for 5 min at room temperature. After three washes, the coverslips were mounted on glass slides with 90% (v/v) glycerol in PBS. The samples were analysed by CLSM, using a Zeiss LSM 900 instrument, Zen Blue software and ImageJ (Schneider et al., 2012).

### Replication Kinetics

MA104 cells (0.7 × 10^6^ cells/ml) were seeded in 25-cm^3^ flasks (Thermo Fischer Scientific) and allowed to grow to 80% confluence before being supplemented with 50 μM GLA 24 h prior to infection or rinsed three times with PBS and incubated with either 25 μM indomethacin, 5 μM celecoxib, 2.5 μM SC-560 or 25 nM CAY10502 in serum-free DMEM for 1 h prior to infection. The aforementioned was repeated every 4 h till the completion of the experiment. For addback experiments, 0.1 μM of exogenous PGE_2_ was added to the cells directly after addition of inhibitors. SA11 was activated for 1 h at 37°C with 10 μg of trypsin/ml, diluted in serum-free DMEM Subsequently, MA104 cells were infected with SA11 at a MOI of 5, both in the presence and absence of GLA or the inhibitors. For the replication studies, time point 00:00 is defined as the initial viral inoculum, while time point 00:30 is cells that were washed with PBS and subsequently freeze-thawed. After infection for 1 h at 37°C, the inoculum was removed and replaced with serum-free DMEM containing 1 μg of trypsin/ml. SA11 was harvested from MA104 cells by three cycles of freeze-thawing, whereafter cellular debris was removed by centrifugation for 10 min at 4,000 × *g*. The supernatant was used to determine viral RNA yield (qRT-PCR) and infectious viral yield (TCID_50_).

### RNA Isolation and RT-qPCR

In order to evaluate the production of VP6 transcripts, the VP6 coding sequence was inserted into the pTZ57R/T cloning vector (Thermo Fischer Scientific). Positive-sense single-stranded RNA was transcribed using the TranscriptAid T7 High Yield Transcription Kit (Thermo Fischer Scientific), using 1 μg XbaI-linearized (Thermo Fischer Scientific) plasmid DNA as template, followed by incubation with DNaseI (Thermo Fischer Scientific) at 37°C for 20 min. *In vitro* synthesized RNA was purified using Trizol reagent (Thermo Fischer Scientific) according to the manufacturer’s instructions and quantified with a Qubit RNA BR kit (Thermo Fischer Scientific). RNA from RV-infected MA104 cells, in the presence or absence of PGE_2_ biosynthesis inhibitors, was semi-purified by 35% (w/v) sucrose cushion ultracentrifugation at 6 h post-infection (Arnold et al., 2009). RNA was extracted from the semi-purified virus by using Trizol reagent according to the manufacturer’s instructions.

Transcribed RNA was used in 2-fold serial dilutions to generate standard curves for determination of the assay efficiency. The Qiagen Rotor-Gene Q System with the Luna^®^ Universal Probe One-Step RT-qPCR Kit (New England Biolabs) was used for RT-qPCR according to the manufacturers protocol using the following primers: cDNA primer (5′-AGGAACGGAATTGCACCT-3′; Integrated DNA Technologies), qPCR forward (5′-CTGGATTTGACTACTCATG-3′) reverse (5′-CGTCTGGTAGAAGAGTTA-3′) and probe (5′-/56-FAM/AACGCACCAGCCAATATACAA-3′; Integrated DNA Technologies). Cycling conditions were 55°C for 10 min, then 95°C for 1 min, followed by 40 cycles of 95°C for 10 s and 60°C for 30 s. Data were analysed with Q-Rex Software Version 1.1.

### Attachment and Internalisation Assay

The effect of PGE_2_ on RV attachment and internalisation was determined using a modified protocol for influenza A virus (Pohl and Stertz, 2015). Twenty-four-well plates were seeded with MA104 cells (0.2 × 10^6^ cells/ml) and supplemented and/or treated with inhibitors/exogenous PGE_2_ as previously described. To determine if the inhibitors of PGE_2_ affected attachment, RV-infected MA104 cells were tagged with FITC anti-RV (Abcam) and anti-DLP (rabbit polyclonal antibody raised against RV double-layered particle; a kind gift from Prof. AC Potgieter, Deltamune, South Africa) at time 0 min. The experimental set consisted of two groups: ‘0 min’ and ‘0 min + anti-DLP’. Rotavirus (MOI = 10) was allowed to cold-bind to supplemented and/or treated MA104 cells for 1 h on ice and then washed three times with ice-cold PBS. The ‘0 min’ and ‘0 min + anti-DLP’ samples were fixed in 4% (v/v) paraformaldehyde for 10 min at room temperature, washed three times and stored at 4°C until analysis.

To determine if the inhibitors of PGE_2_ affected internalisation, RV-infected MA104 cells were again tagged with FITC anti-RV and anti-DLP at 30 min. After the incubation on ice, the ‘30 min’ and ‘30 min + anti-DLP’ samples were washed and PBS containing 2% (w/v) BSA and 1 μg/ml trypsin was added to the MA104 cells, which were then incubated for 30 min at 37°C, fixed and washed three times. Following fixation, all samples were permeabilised with PBS containing 0.5% (v/v) Triton X-100 for 5 min at room temperature.

All the permeabilised cells were incubated for 1 h at 37°C in PBS containing 2% BSA and 1:500 FITC anti-RV. After staining with FITC Anti-RV, cells were washed three times and all the cells were incubated for 1 h at 37°C in PBS containing 2% BSA and 1:500 anti-DLP. Cells were again washed and incubated with 1:500 Alexa Fluor 532 (Thermo Fischer Scientific). Cells were extensively washed and detached with StemPro^™^ Accutase^™^ (Thermo Fischer Scientific) for 30 min at 37°C before analyses on the BD Accuri C6 Plus instrument.

### Statistical Analysis

Each assay was carried out at least in triplicate (biological/independent replicates) on separate days. Each repeat was analysed in duplicate (*n* = 3). Data are presented as means ± standard error of the mean. For statistical analysis, two-way ANOVA was performed using a Tukey-Kramer test in GraphPad Prism version 3.00 for Windows (GraphPad Software, San Diego, Calif.). In all tests, *p* < 0.05 was considered statistically significant.

## Results

### Rotavirus Infection Decreases the Relative Percentage of γ-Linolenic and Arachidonic Acid

**Figure 1:**
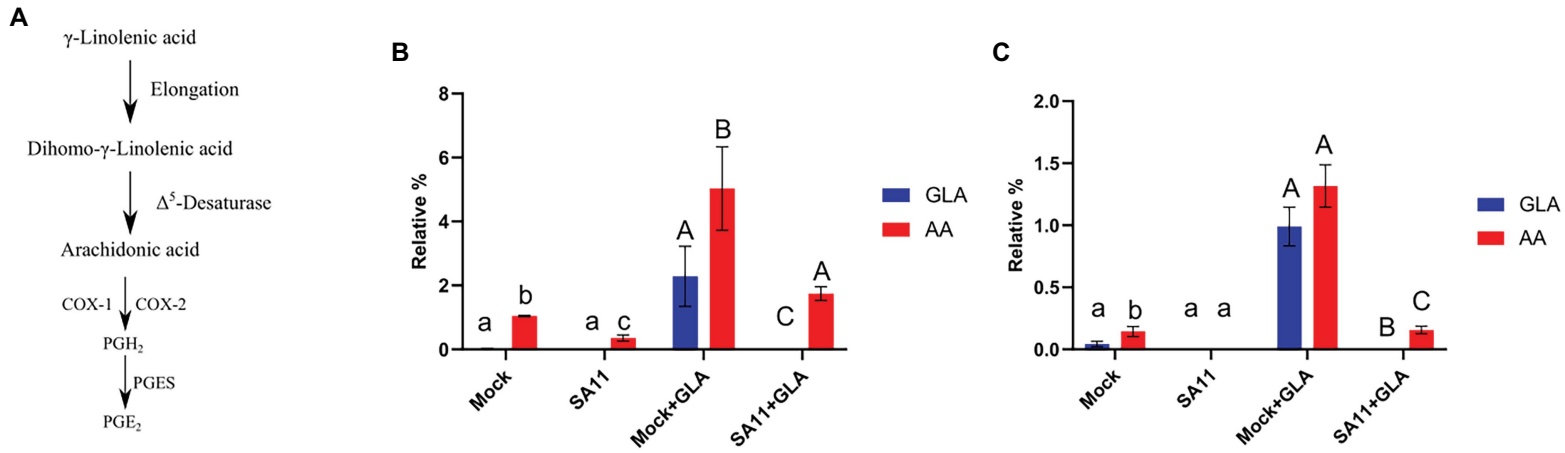
Conversion of GLA to AA at 16 h post-infection. **(A)** γ-Linolenic acid (GLA) is converted to dihomo-γ-linolenic acid by elongases which is subsequently converted to arachidonic acid (AA) *via* Δ^5^-desaturase. Finally, AA is converted to PGE_2_ by either COX-1/2 and PGES (Funk, 2001). **(B)** The effect of rotavirus on the relative percentage of GLA (blue) and AA (red) in the neutral lipid fraction **(B)** and phospholipid fraction **(C)** was evaluated in unsupplemented (mock; SA11) and supplemented (mock + GLA; SA11 + GLA) MA104 cells. Error bars indicate the standard error of the mean (*n* = 3). Lowercase and uppercase letters indicate a significant difference (*p* < 0.05) compared to the control. Cyclooxygenases (COX1/2), prostaglandin E_2_ synthase (PGES); and prostaglandin E_2_ (PGE_2_).

During PGE_2_ biosynthesis, AA is liberated by phospholipase A_2_ (Kudo and Murakami, 2002), and converted to PGH_2_, by either COX-1 (in a continuous manner) or COX-2 (in a stimuli-dependent manner; Park et al., 2006). In the final step of PGE_2_ synthesis, PGH_2_ is converted to PGE_2_ by one of three prostaglandin E synthetases (Figure 1A). Arachidonic acid can also be synthesized in cells by the addition of two-carbons to GLA, to form dihomo-γ-linolenic acid, followed by the introduction of a double bond (Lagarde et al., 2013; Figure 1A). As expected, it was found that GLA supplementation of MA104 cells causes a significant increase in the relative percentages of GLA and AA in the neutral (*p* = 0.004; *p* = 0.0042, respectively; Figure 1B) and phospholipid fractions (*p* = 0.026; *p* = 0.016, respectively; Figure 1C). Interestingly, when both the unsupplemented and supplemented MA104 cells were infected with SA11, the relative percentages of both GLA and AA were significantly reduced in both fractions (*p* = 0.04; *p* = 0.0028, respectively), compared to the uninfected cells. The phenomenon is more pronounced in the phospholipid fraction with an 8× decrease compared to a 3× decrease in neutral lipid fraction.

### Rotavirus Infection Stimulates PGE_2_ Production in a Dose-Dependent Manner

**Figure 2:**
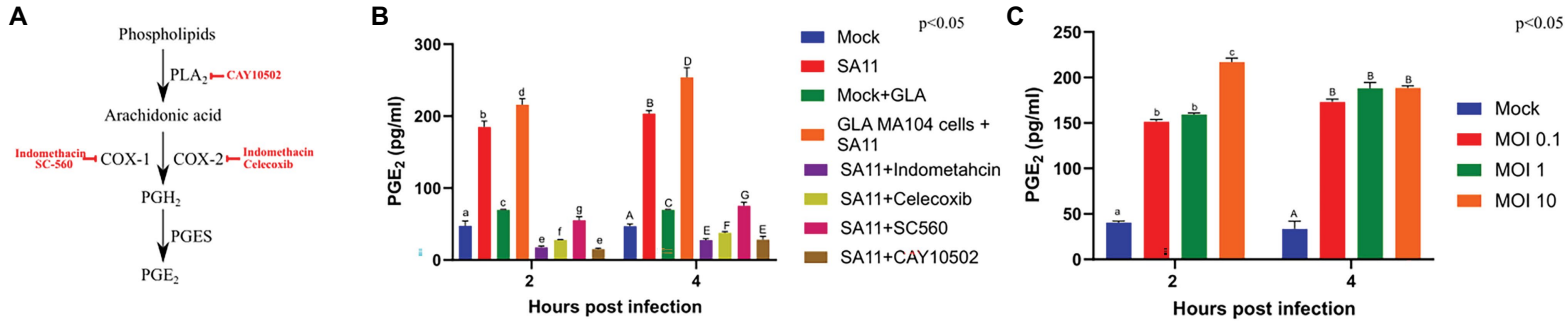
Rotavirus increases the concentration of prostaglandin E_2_ in MA104 cells in a dose- and time-dependent manner. **(A)** CAY10502 inhibits the releases of AA from phospholipids, while indomethacin non-specifically inhibits both COX-1 and COX-2. In contrast, SC-560 specifically inhibits COX-1 and celecoxib specifically inhibits COX-2. **(B)** The amount of PGE_2_ produced during rotavirus (SA11) infection (MOI = 5) as determined by ELISA was compared to uninfected MA104 cells (mock), in the presence and absence of GLA and inhibitors of the PGE_2_-biosynthetic pathway at 2 and 4 h post-infection. **(C)** The amount of PGE_2_ produced at increasing multiplicities of infection (MOI) at 2 and 4 h post-infection. Error bars indicate the standard error of the mean (*n* = 3). Lowercase and uppercase letters indicate a significant difference (*p* < 0.05) compared to the control. Cyclooxygenases (COX1/2), phospholipaseA_2_ (PLA_2_), prostaglandin E_2_ synthase (PGES); and prostaglandin E_2_ (PGE_2_).

The decrease in both the relative percentages of GLA and AA in GLA-supplemented, infected cells could possibly be due to the conversion of AA to PGE_2_, which is rapidly produced in response to stimuli (Berenbaum, 2000). Therefore, the concentration of PGE_2_ produced by cells during viral infection was determined. The supplementation of MA104 cells with GLA in the absence of virus caused an increase in PGE_2_, as measured by ELISA, at both 2 and 4 h post-infection, indicating a shift in the baseline production of PGE_2_ in GLA-supplemented cells (Figure 2). Rotavirus infection of unsupplemented cells also caused a significant increase in PGE_2_ production at both time points (*p* = 0.00004; *p* = 0.00008, respectively). It should also be noted that the concentration of PGE_2_ increased from 2 to 4 h post-infection regardless of supplementation, but the effect is more significant in supplemented MA104 cells (increase of ~38 pg/ml vs. ~18.5 pg/ml increase in unsupplemented cells; *p* = 0.047).

Figure 2A indicates where each of the inhibitors act in the PGE_2_ biosynthesis pathway. Treatment of infected MA104 cells with inhibitors of PGE_2_ biosynthesis decreased the levels on PGE_2,_ with the indomethacin (*p* = 0.0008) and CAY10502 (*p* = 0.0004) having the biggest effect followed by celecoxib (*p* = 0.00009) and SC560 (*p* = 0.0007; Figure 2B). To evaluate if PGE_2_ production is viral load dependent, PGE_2_ production was evaluated at different MOIs using ELISA (Figure 2C). The production of PGE_2_ by mock-infected cells remained constant from 2 to 4 h post-infection, while there was a steady increase in the amount of PGE_2_ produced in cells infected at a MOI of 0.1 and 1. Interestingly, the production of PGE_2_ increased rapidly in cells infected with a MOI of 10, appearing to peak at 2 h post-infection. The difference in PGE_2_ levels at different MOIs shows that SA11 modulates PGE_2_ concentration in a time- and viral dose-dependent manner. Chromatograms obtained from LC-MS/MS showed that all five transitions of PGE_2_ were present in all the infected samples, thus authenticating its presence (Supplementary Figure S2). The discrepancy between ELISA and LC-MS/MS data for the mock control could be due to the ability of the ELISA to cross-react with PGE_2_-ethanolamide and/or PGE_2_-1-glycerylester or the inherently low concentration of PGE_2_ in unstimulated cells (Funk, 2001). In addition, the discrepancy could also be attributed to the higher sensitivity of the ELISA compared to LC-MS.

### Prostaglandin E_2_ and Rotavirus Co-localise

**Figure 3:**
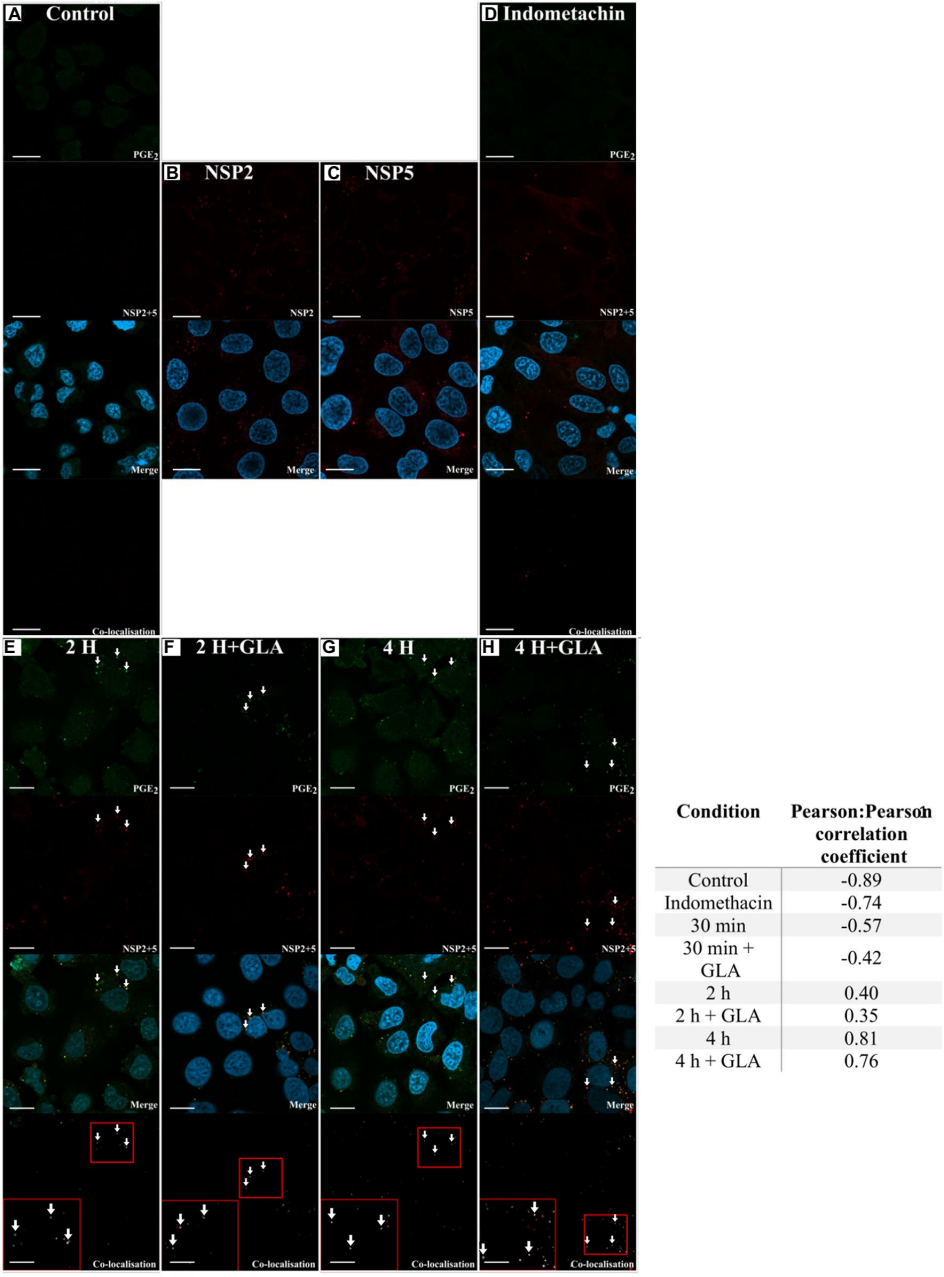
Laser scanning microscopy images showing co-localisation between RV and PGE_2_. The presence of co-localisation (white arrows) between PGE_2_ (green) and RV NSP2 and NSP5 (red) was evaluated using Coloc2 in ImageJ. Blue DAPI-staining indicates cell nuclei. **(A)** The no-virus control showed no detection of RV. The ability of the **(B)** anti-NSP2 and **(C)** anti-NSP5 antibodies to detect RV was independently assessed. **(D)** The required inclusion of indomethacin shows no detection of PGE_2_. After 2 h post-infection, NSP2 and NSP5 were detected in both **(E)** GLA unsupplemented and **(F)** supplemented cells, with co-localisation with PGE_2_ first detected as suggested in the merge and confirmed in the co-localisation panels. Co-localisation increases at 4 h post-infection both in the **(G)** absence or **(H)** presence of GLA. Small red squares in the co-localisation panels indicate the area that was magnified and shown in the large red square in the same panel. Pearson:Pearson correlation coefficients of confocal microscopy images as determined by ImageJ are shown in the adjacent table (Schneider et al., 2012). Values close to −1 indicate no co-localisation with values close to 0 indicating random co-localisation, while values close to 1 indicate co-localisation with a high degree of certainty. Prostaglandin E_2_(PGE_2_). White arrows indicate co-localisation. Scale bar 10 μM.

Lipid droplets are known sites for PGE_2_ synthesis (Accioly et al., 2008; Bozza et al., 2011) and RV viroplasms are known to associate with LDs (Cheung et al., 2010). Therefore, to assess if there is co-localisation between viroplasms and PGE_2_, we targeted NSP5 and NSP2 to visualise viroplasms. Both anti-NSP2 and anti-NSP5 antibodies were able to independently detect viroplasms (Figures 3B,C). As expected, cells treated with indomethacin showed no co-localisation due to reduced PGE_2_ production (Figure 3D). Indomethacin is a non-specific inhibitor of both COX-1 and COX-2 (Mitchell et al., 1993) and is included in the EicosaCell procedure as negative control to inhibit PGE_2_ production (Bandeira-Melo et al., 2011). Co-localisation, albeit at a relative low level, was observed 2 h post-infection in both unsupplemented (Figure 3E) and GLA-supplemented cells (Figure 3F). This observation is confirmed by the Pearson:Pearson correlation coefficient (0.40; Figure 3). An increase in co-localisation was observed 4 h post-infection (Figures 3G,H) with the Pearson:Pearson correlation coefficient reaching a maximum of 0.81 (Figure 3).

### Rotavirus Replication Is Enhanced by Changes in Cellular Lipids

**Figure 4:**
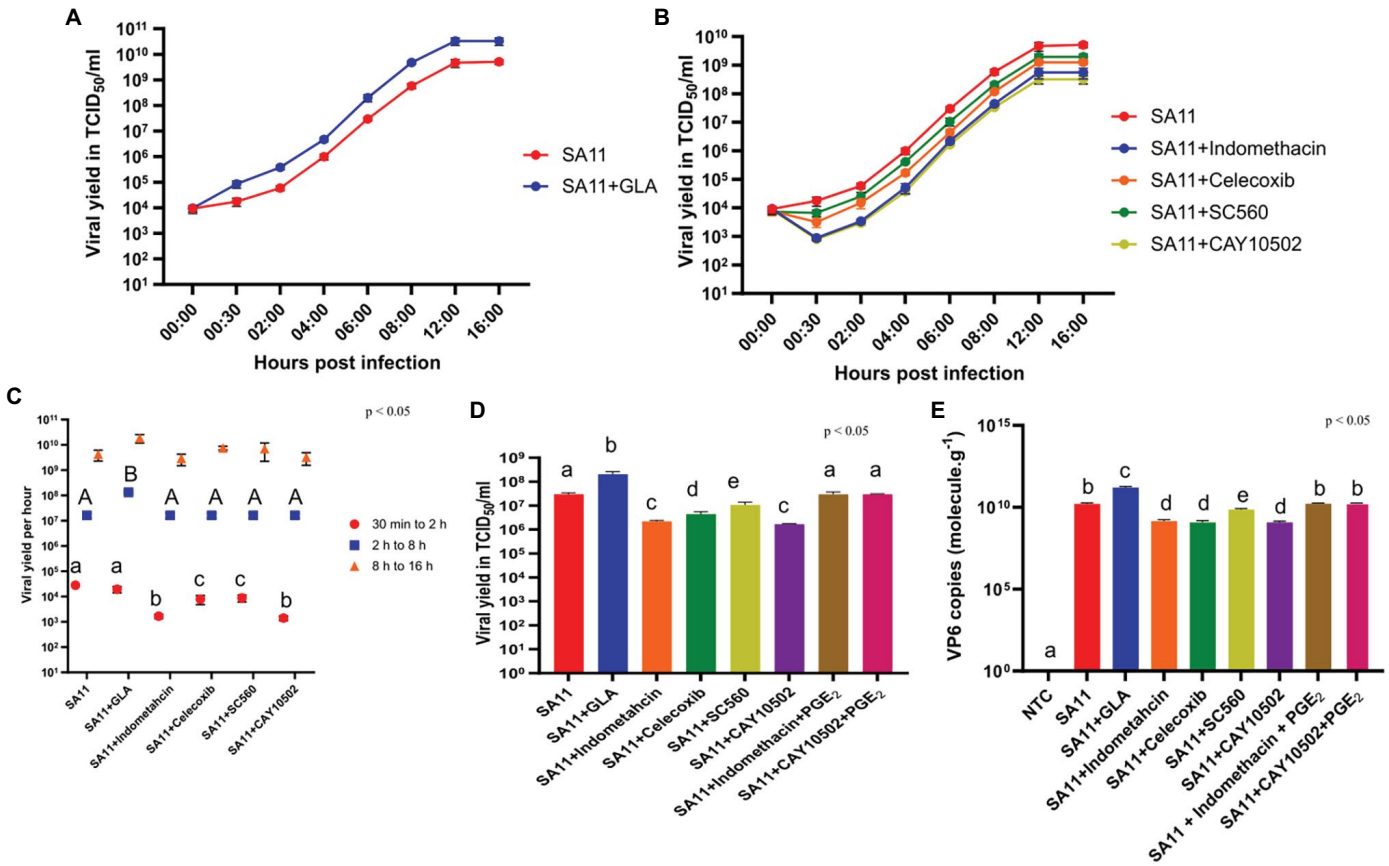
GLA supplementation and treatment with inhibitors of PGE2 biosynthesis affect RV replication. **(A)** Rotavirus yield determined by TCID_50_ was compared to SA11-infected MA104 cells, in the presence and absence of GLA and **(B)** inhibitors of the PGE_2_-biosynthetic pathway at several time points post-infection. **(C)** The replication rate of rotavirus as determined by calculating the slope between 30 min and 2 h (red), 2 h and 8 h (blue) or 8 h and 16 h (orange), was compared to SA11-infected MA104 cells, in the presence and absence of GLA and inhibitors of the PGE_2_-biosynthetic pathway. **(D)** Rotavirus yield as determined by TCID_50_ and **(E)** copy number of VP6 as determined by RT-qPCR was compared to SA11-infected MA104 cells, in the presence and absence of GLA and inhibitors of the PGE_2_-biosynthetic pathway at 6 h post-infection. Error bars indicate the standard error of the mean (*n* = 3). Lowercase and uppercase letters indicate significant difference (*p* < 0.05) compared to the control.

In order to determine if the modulation of AA and GLA and the subsequent increase in PGE_2_ had any effect on viral replication, we employed replication kinetics to determine the effect of GLA supplementation on the replication of SA11. The supplementation of MA104 cells with GLA increased the viral yield at 30 min (Figure 4A). This initial effect caused an approximate 1 log increase in the overall viral yield after the 16-h period, compared to the unsupplemented MA104 cells. The addition of several PGE_2_ biosynthetic inhibitors affected the yield of SA11 during the 16-h period (Figure 4B). Treatment of MA104 cells with indomethacin (non-specific COX inhibitor) caused the largest decrease in viral yield at 30 min, followed by CAY10502 (cPLA_2_ inhibitor), celecoxib (COX-2-specific inhibitor) and SC-560 (COX-1-specific inhibitor). The rate of replication between different time points is shown in Figure 4C. Interestingly, the supplementation of MA104 cells with GLA only increased the rate of replication significantly between 2 and 8 h post-infection, while the rate of replication remained comparable to the control at 30 min to 2 h as well as between 8 and 16 h post-infection. Similarly, the treatment of MA104 cells with PGE_2_ biosynthesis inhibitors significantly decreased the rate of replication between 30 min to 2 h, while no significant difference was observed at the other time points.

To ensure that the effect of the inhibitors on viral yield was due to their inhibitory effect on PGE_2_ biosynthesis, viral yield was determined with indomethacin in the presence of 0.1 μM PGE_2_ at 6 h post-infection (Figure 4D). In addition, RNA production was determined with RT-qPCR, targeting the genome segment encoding VP6, and calculating the relative copy number in GLA-supplemented MA104 cells and in cells treated with the different PGE_2_ biosynthesis inhibitors (Figure 4E). GLA supplementation of MA104 cells and subsequent infection with SA11 significantly increased both viral yield and the copy number of the VP6 genome segment. In contrast, treatment of SA11 infected MA104 cells with COX inhibitors significantly decreased the viral yield and copies of the VP6 genome segment. The viral yield was most affected by indomethacin and CAY10502, followed by celecoxib and SC-560. Interestingly, indomethacin, CAY10502 and celecoxib appear to have the same decreasing effect on the copy number of the VP6 genome segment, while SC-560 has the least potent effect. The addition of PGE_2_ to MA104 cells treated with indomethacin or CAY10502, restored both the viral yield and copies of VP6 to control levels, indicating that PGE_2_ is important for SA11 replication in MA104 cells.

### Cyclooxygenase Inhibition and GLA Supplementation Affect Rotavirus Internalisation

**Figure 5:**
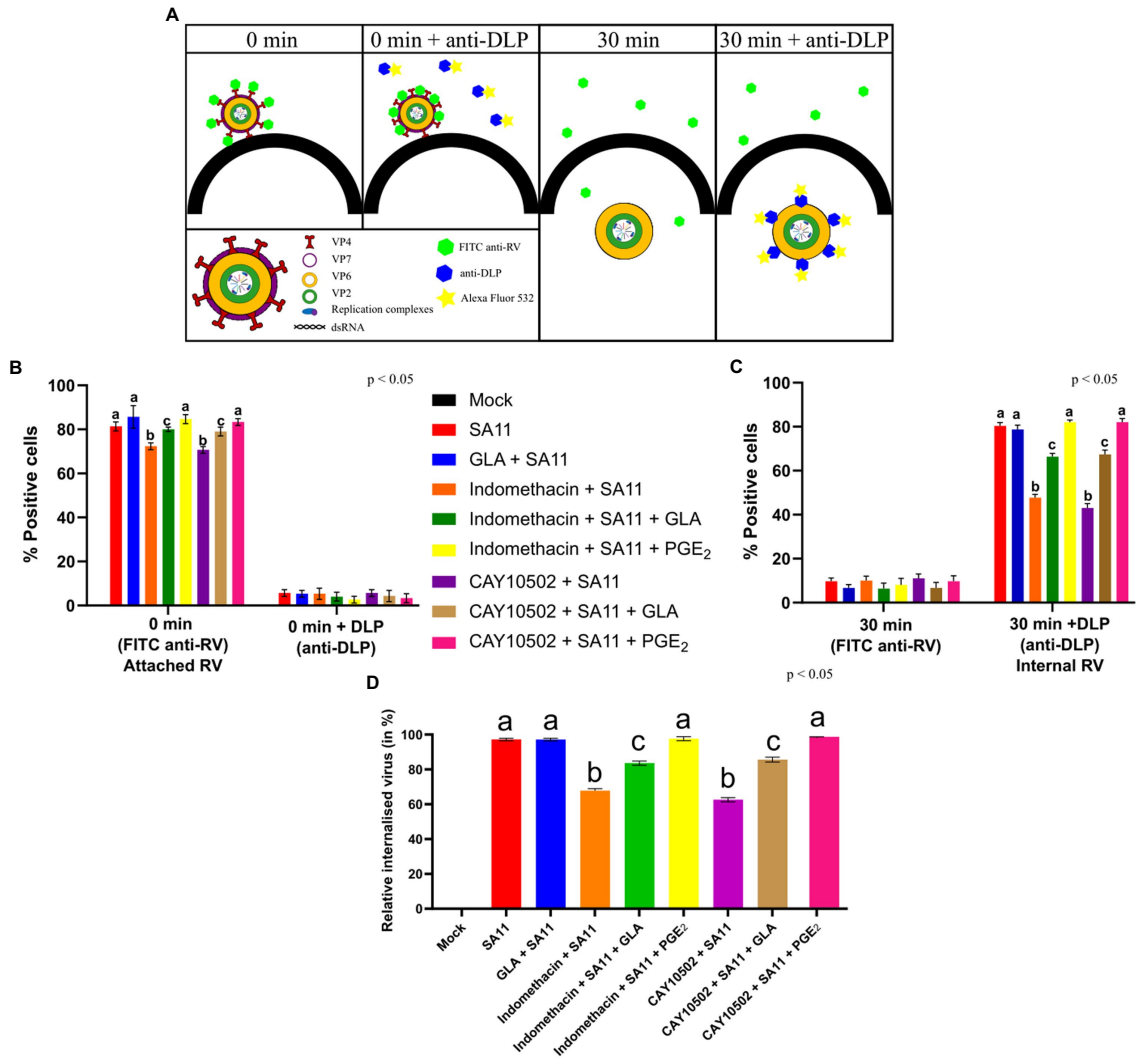
Inhibition of PGE2 biosynthesis affects RV attachment and internalisation. **(A)** At time zero, RV at an MOI = 10 is cold-bound to MA104 cells and is visualised by anti-RV-FITC labelling, which detected the outer capsid protein VP7. When labelling with anti-double-layered particle (DLP) is applied, virus at the cell surface cannot be detected as the antibodies target the middle-layer protein, VP6, which is only accessible after the outer layer has been shed. Thus, the signal intensity of 0 min + anti-DLP is strongly reduced compared to 0 min + anti-RV due to the inability to detect VP6. When the temperature is increased, RV is internalised and the loss of the outer capsid allows for the detection of VP6, i.e., DLPs, to be accessible for staining. **(B)** Graph showing a higher positive detection of anti-RV-FITC compared to anti-DLP at 0 min. **(C)** Graph showing a higher positive detection of anti-DLP compared to anti-RV-FITC at 30 min. **(D)** Relative percentage of internalised virus from untreated and inhibitor-treated cells at 30 min post-infection is calculated by dividing the relative internalised RV (30 min) **(C)** by relative attached RV (0 min) **(B)**. Error bars indicate the standard error of the mean (*n* = 3). Lowercase letters indicate significant differences (*p* < 0.05) compared to the control.

Due to the effect of GLA supplementation and inhibition of PGE_2_ biosynthesis observed during the initial stages on SA11 replication, we used flow cytometry to determine if PGE_2_ and GLA supplementation play a role during the attachment and/or internalisation of SA11. Figure 5A shows the principle of the internalisation and attachment assay. Briefly, RV was allowed to cold-bind to MA104 cells. The virus was then subsequently tagged with anti-RV-FITC, which detects the outer capsid protein VP7, indicating the percentage of attached virus. When RV is internalised and released from the endosomes, the shedding of the outer layer exposes VP6 and allows for tagging with anti-DLP (targeting the outer VP6-layer of the DLP) and Alexa Fluor 532, quantifying intracellular RV. Internalisation was calculated by dividing relative percentage intracellular RV (30 min + DLP) by relative percentage attached RV (0 min). Results compiled from raw flow cytometry data (Figure 5B) indicate that the supplementation of MA104 cells with GLA had no significant effect (*p* = 0.067) on SA11 attachment, but the treatment of MA104 cells with indomethacin (*p* = 0.008) and CAY10502 significantly decreased (*p* = 0.019) the percentage of attached SA11. The addition of exogenous PGE_2_ to the inhibitor-treated cells fully restored the percentage of attached SA11. Interestingly, the supplementation of inhibitor-treated cells with GLA only partially restored the percentage of attached SA11, indicating that the amount of PGE_2_ produced from GLA may not be sufficient to completely restore attachment. Figure 5C shows the amount of RV that was internalised after 30 min. Although the effect of PGE_2_ inhibition on attachment is significant, the effect on internalisation is much more pronounced (Figure 5D). Treatment of MA104 cells with either indomethacin (*p* = 0.0014) or CAY10502 (*p* = 0.003) decreased the percentage of internalised SA11, while the addition of exogenous PGE_2_ fully restored the percentage of internalised SA11 and GLA supplementation only partially restored the percentage of internalised SA11. This shows a role of PGE_2_ during both the attachment and internalisation of SA11.

## Discussion

Lipids and in particular LDs are crucial for RV replication, as chemical compounds affecting the integrity of LDs can disrupt viroplasms and subsequently, viral RNA replication and progeny production (Cheung et al., 2010; Crawford and Desselberger, 2016). Previously, it was shown that supplementation of cells with saturated fatty acids increased RV yield (Superti et al., 1995). Furthermore, studies have shown that PGE_2_ is elevated during both *in vitro* (Rossen et al., 2004) and *in vivo* (Zijlstra et al., 1999) RV infection, with the inhibition of PGE_2_ biosynthesis, decreasing the duration of diarrhoea (Yamashiro et al., 1989).

Viruses have been shown to modulate lipid metabolism, composition and/or signalling to ensure successful viral entry, replication, assembly and/or secretion (Heaton and Randall, 2011). We show that RV depletes both AA and GLA during infection of MA104 cells, indicating the ability of the virus to modulate the metabolism of these cellular fatty acids, leading to increased PGE_2_ production. Although AA is present in both the neutral lipid and phospholipid fractions of LDs, it is speculated that AA found in the neutral lipid fraction only replenishes the AA levels in the phospholipid fraction when levels are depleted due to eicosanoid production (Bozza et al., 2011). Prostaglandin E_2_ is the most abundant prostanoid and exerts homeostatic, pro- and, in certain cases, anti-inflammatory effects in host cells (Park et al., 2006). Thus, it is not surprising that PGE_2_ plays a role in the pathogenesis of several viruses, including human immunodeficient virus, influenza A and herpes simplex virus (Sander et al., 2017). Along with the aforementioned viruses, RV has been shown to increase PGE_2_ in both *in vitro* (Rossen et al., 2004) and *in vivo* studies (Zijlstra et al., 1999), while increased levels of PGE_2_ have also been found in the stool of children infected with RV (Yamashiro et al., 1989). In these studies, an increase in PGE_2_ was accompanied by an increase in RV yield as well as severity and longevity of gastroenteritis.

In concurrence with previous studies (Yamashiro et al., 1989; Zijlstra et al., 1999; Rossen et al., 2004), we have also shown that SA11 increases the concentration of PGE_2_ and that the GLA supplementation of MA104 cells leads to a further increase in the concentration of PGE_2_. These increases in PGE_2_ levels are time and viral dose-dependent and coincide with the decrease in AA and GLA during RV infection. Supplementation of MA104 cells with GLA significantly increased the rate of RV replication only between 2 and 8 h post-infection, with no significant effect on the rate of replication early (30 min to 2 h) or late (8–16 h) during infection. This indicates that GLA has no effect on early replication but may play a role later during replication, probably during the formation of viroplasms, as these occur approximately 2 h post-infection (Carreño-Torres et al., 2010), by increasing the amount of LD available as scaffolds (Exner et al., 2019).

It is well known that PGE_2_ is produced in LDs in response to external stimuli, such as viral infections (Accioly et al., 2008; Bozza et al., 2011). Furthermore, during RV, replication LDs serve as scaffolds for the formation of viroplasms (Cheung et al., 2010). Viroplasms consist of several viral proteins, with NSP2 and NSP5 being essential for their formation. Viroplasms start to form at 2 h post-infection and increase in number as infection progresses (Carreño-Torres et al., 2010; Contin et al., 2010). We show that co-localisation does occur between NSP2 and NSP5 and PGE_2_ at 2 and 4 h post-infection. Interestingly, we show that co-localisation between NSP2 and NSP5 and PGE_2_ increases as infection progresses and could be due to an increase in both the number of viroplasms that are formed and the increase in PGE_2_ levels as shown by ELISA.

The inhibition of COXs has shown that these enzymes are essential during replication of several viruses (Steer and Corbett, 2003). Although, indomethacin is a well-known non-selective inhibitor of COX-1 (IC_50_ = 1.67 μM) and COX-2 (IC_50_ = 24.6 μM) it has several off-targets effects including the inhibition of phospholipase A_2_ (Kaplan et al., 1978). We therefore employed more specific inhibitors for COX-1 (SC-560, IC_50_ = 9 nM; Smith et al., 1998), COX-2 (celecoxib, IC_50_ = 5 nM; Uddin et al., 2003) and phospholipase A_2_ (CAY10502, IC_50_ = 4.3 nM; Ludwig et al., 2006). It should however be noted that celecoxib has been shown to inhibit several other enzymes, such as carbonic anhydrases (IC_50_ = 16 nM), phosphoinositide-dependent kinase-1 (IC_50_ = 48 μM) and sarcoplasmic/ER calcium ATPase (Schönthal, 2007). It is therefore possible that at the concentration of celecoxib (5 μM) used in this study, carbonic anhydrases could also have been inhibited. However, the results for indomethacin and celecoxib correlate with that for the COX-1 (SC-560) and phospholipase A_2_ (CAY10502) inhibitors. Phospholipase A_2_ is also the rate-limiting enzyme during the biosynthesis of PGE_2_ (Funk, 2001). In addition, the effects on RV replication and entry were neutralised when exogenous PGE_2_ was added. Combined, these results therefore suggest that the inhibition of PGE_2_ affects RV replication.

Treatment of MA104 cells with inhibitors of PGE_2_ biosynthesis has detrimental effects on viral yield and on the rate of replication between 30 min and 2 h post-infection. This is in concurrence with Rossen et al. (2004), who determined that RV replication is negatively affected by treatment with PGE_2_ biosynthesis inhibitors and indicated a role of PGE_2_ during early RV replication. However, in contrast to their results, showing no effect on total RNA levels from RV NSP4 and VP4 or on the ssRNA and dsRNA derived from VP4, our results show that all the inhibitors had a detrimental effect on the RNA levels of the VP6 genome segment. The discrepancy could be due to differences in experimental procedures used as well as the time point measured. In the current study, purified viral RNA was used in a RT-qPCR analysis, compared to a semiquantitative RT-PCR evaluation of total, single-stranded and double-stranded RNA by Rossen et al. (2004). The authors also determined RNA levels at 15 h post-infection, while we determined RNA levels at 6 h post-infection. The addition of exogenous PGE_2_ to inhibitor-treated cells restored both the viral yield and RNA levels of VP6. Similar to the findings by Rossen et al. (2004), we show that there is no significant effect of PGE_2_ biosynthetic inhibitors on late stage (8–16 h) RV replication.

Due to the effect of PGE_2_ biosynthesis inhibitors during early infection (0 min to 30 min) and in concurrence with data from Rossen et al. (2004), which show that the inhibitors are most potent when added at time point zero, we investigated RV attachment and internalisation in the presence of PGE_2_ biosynthesis inhibitors and GLA supplementation. We show that the treatment of MA104 cells with inhibitors of PGE_2_ significantly decreases the amount of RV that is internalised. The effect of early and non-specific inhibition of PGE_2_ indicates a role for PGE_2_ during the early phase of SA11 infection. In addition, the significant difference (*p* < 0.05) in viral yield with cells treated with celecoxib and SC560 possibly indicates a more pronounced role for induced PGE_2_ (*via* COX-2), consistent with data from Rossen et al. (2004). While the addition of exogenous PGE_2_ restored the levels of RV that is internalised, supplementation with GLA only partially restored internalisation. In addition, the supplementation of MA104 cells with GLA does not significantly affect RV attachment, which is in concurrence with Superti et al. (1995) that showed the effect of fatty acid supplementation on viral replication did not affect attachment.

## Conclusion

We show that RV can modulate host lipids to the benefit of its replication cycle. The supplementation of MA104 cells with GLA increases the production of PGE_2_, which in turn enhances RV attachment and internalisation. Exactly how PGE_2_ affects these early steps in RV replication remains unknown. It is possible that PGE_2_ promotes Ca^2+^-mediated epithelial barrier disruption (Martién-Venegas et al., 2006; Rodríguez-Lagunas et al., 2010) or that PGE_2_ creates a pro-inflammatory environment that benefits viral replication (Sander et al., 2017). Interestingly, Cheng et al. (2015) showed that the internalisation of bovine ephemeral fever virus (BEFV) through clathrin-mediated endocytosis required activation of several pathways, including COX-2-mediated PGE_2_/Prostaglandin receptors receptor signalling. They showed that BEFV activates the Src-JNK-AP1 and PI3K-Akt-NF-κB signalling pathways, which in turn modulates COX-2 expression and PGE_2_ production. The binding of PGE_2_ to G-protein-coupled E-(EP) prostanoid receptors stimulated Src-JNK-AP1 and PI3K-Akt-NF-κB signalling, promoting viral entry. In addition, Kaposi’s sarcoma-associated herpesvirus, human immunodeficiency virus and human T-lymphotropic virus type 1 have all been shown to utilise PGE_2_/EP receptor-mediated signalling (Dumais et al., 1998; Moriuchi et al., 2001; Paul et al., 2013). Although no direct evidence links RV and PGE_2_/EP signalling, it is known that RV activates both the JNK-AP1 and PI3K-Akt signalling pathways (Holloway and Coulson, 2006; Soliman et al., 2018). The phosphorylated pPI3K, pAkt and pERK interact with V-ATPase, increasing the proton gradient within endosomes resulting in their acidification and release of RV into the cytoplasm. Furthermore, Holloway and Coulson (2006) showed that the activation of JNK and p38 leads to AP-1-driven transcriptional responses, which can influence RV mRNA levels and replication. It should be noted that the activation of JNK was only observed at 2 and 6 h post-infection in Caco-2 and MA104 cells, respectively. It could thus be possible that the increase in PGE_2,_ during RV infection as observed in this study and others, enhances the internalisation of SA11 by clathrin-mediated endocytosis, as several RV strains use clathrin-mediated endocytosis for entry into host cells (Díaz-Salinas et al., 2013; Arias and López, 2021).

This study highlights the importance of lipid modulation during RV infections and indicates the role that biologically important lipid metabolites can play in viral infections, while also identifying possible anti-viral targets.

## Data Availability Statement

The raw data supporting the conclusions of this article will be made available by the authors, without undue reservation.

## Author Contributions

WS, CP, and HO: conceptualisation. WS, GK, and AH: methodology, investigation and data curation. WS: writing-original draft preparation. CP, HO, WS, GK, and AH: writing—review and editing. CP and HO: funding acquisition. All authors contributed to the article and approved the submitted version.

## Funding

The study was supported by the Poliomyelitis Research Foundation (PRF; 19/17) and the Deutsche Forschungsgemeinschaft (DFG; JO369/5-1) to HO and the National Research Foundation (NRF; 115566) to CP. WS is supported by scholarships from the NRF and PRF.

## Conflict of Interest

The authors declare that the research was conducted in the absence of any commercial or financial relationships that could be construed as a potential conflict of interest.

## Publisher’s Note

All claims expressed in this article are solely those of the authors and do not necessarily represent those of their affiliated organizations, or those of the publisher, the editors and the reviewers. Any product that may be evaluated in this article, or claim that may be made by its manufacturer, is not guaranteed or endorsed by the publisher.

## Supplementary Material

Supplementary Figure S1 Cellular toxicity of the inhibitors and DMSO was evaluated with the XTT assay. MA104 cells (4 × 104 cells/ml) were seeded into 96-well plates and allowed to grow to 100% confluence. The inhibitors were added 1 h prior to infection and subsequently every 4 h till the completion of the experiment. **(A)** All inhibitors were added to MA104 cells containing a final concentration of 1% DMSO to ensure the vehicle had no detrimental effect on the cells. Results indicate that 1% decreases cell viability to approximately 92 ± 3%. **(B)** Data obtained from the toxicity assays. Concentrations to be used for the different inhibitors were determined as follows: indomethacin 25 μM (84% ± 0.19), celecoxib 5 μM (85% ± 0.85), SC-560 2.5 μM (83% ± 1.5) and CAY10502 25 nM (93% ± 2.8).

Supplementary Figure S2 Mass spectra showing the five transitions (Multiple reaction monitoring) for each of the samples. Only one transition of PGE2 is present in the mock infections at **(A)** 2 h and **(B)** 4 h. All five the transitions of PGE2 are present in cells infected at a MOI 0.1 **(C,D)** and 1 **(E,F)** at 2 h **(C,E)** and 4 h **(D,F)** post-infection, respectively. Furthermore, all five the transitions of PGE2 are present in cells infected at a MOI of 10 t **(G)** 2 h and **(H)** 4 h post-infection. Due to very low signal to noise ratios, the LC-MS/MS data were only used to verify the presence of PGE2. Peaks show the relevant transitions 351.17 > 315.2; 351.17 > 271.2; 351.17 > 333.3; 351.17 > 189.0; and 351.17 > 235.1. Prostaglandin E2 (PGE2).
