# Corrigendum: Rotavirus-Mediated Prostaglandin E_2_ Production in MA104 Cells Promote Virus Attachment and Internalisation, Resulting in an Increased Viral Load

**DOI:** 10.3389/fphys.2022.901082

**Published:** 2022-04-19

**Authors:** Willem J. Sander, Gabré Kemp, Arnold Hugo, Carolina H. Pohl, Hester G. O'Neill

**Affiliations:** ^1^ Department of Microbiology and Biochemistry, University of the Free State, Bloemfontein, South Africa; ^2^ Department of Animal Science, University of the Free State, Bloemfontein, South Africa

**Keywords:** rotavirus, viroplasm, prostaglandin E_2_, fatty acid supplementation, internalisation, attachment, lipid droplets

In the original article, there was a mistake in Figure 5A as published. In the figure, the rotavirus outer capsid proteins are labeled incorrectly. The spike protein is VP4 and the outer layer protein, VP7, not VP7 and VP4, respectively, as in the published article. The corrected Figure 5A appears below.

**FIGURE 5:**
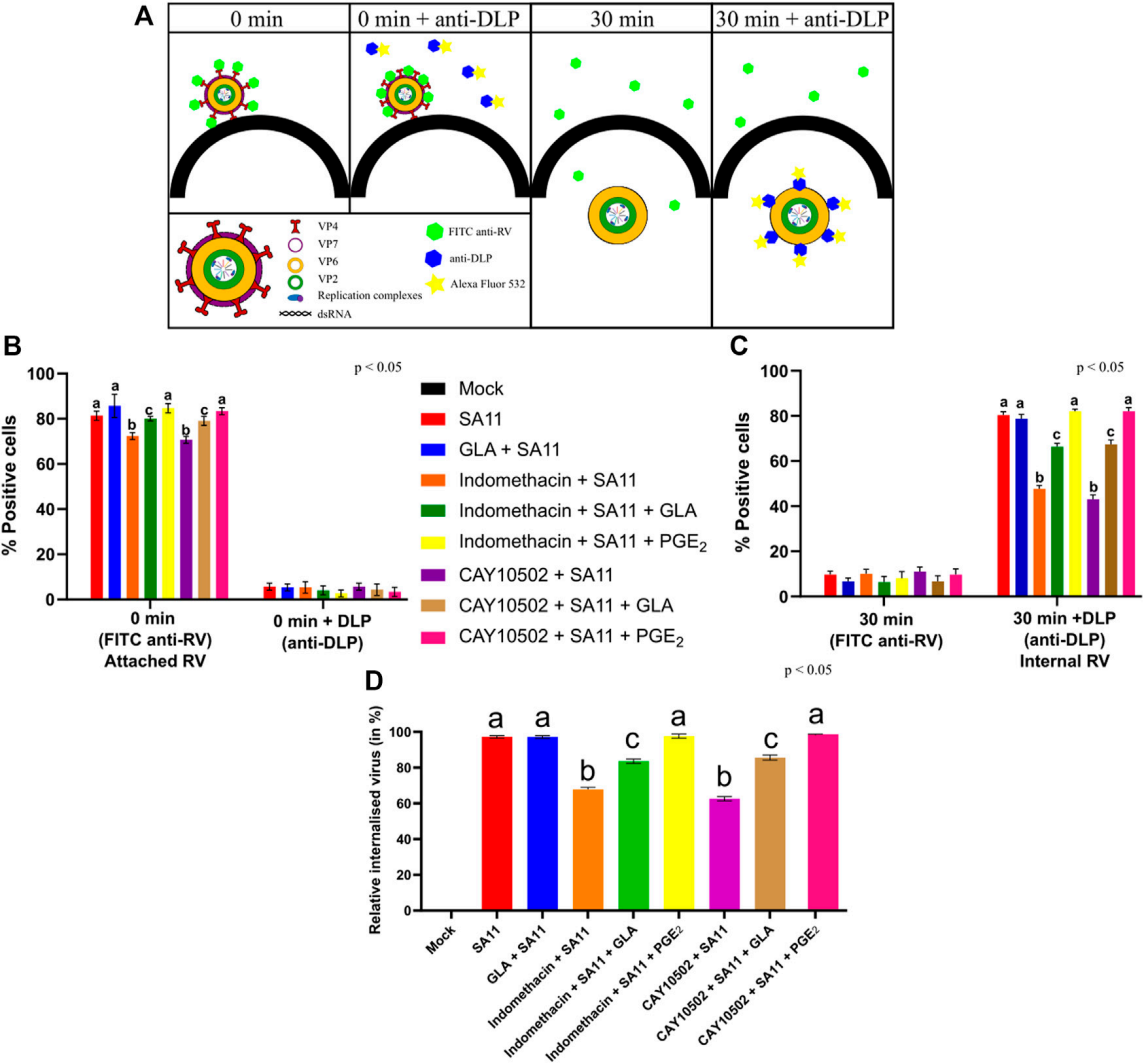
Inhibition of PGE2 biosynthesis affects RV attachment and internalisation. **(A)** At time zero, RV at an MOI = 10 is cold-bound to MA104 cells and is visualised by anti-RV-FITC labelling, which detected the outer capsid protein VP7. When labelling with anti-double-layered particle (DLP) is applied, virus at the cell surface cannot be detected as the antibodies target the middle-layer protein, VP6, which is only accessible after the outer layer has been shed. Thus, the signal intensity of 0 min + anti-DLP is strongly reduced compared to 0 min + anti-RV due to the inability to detect VP6. When the temperature is increased, RV is internalised and the loss of the outer capsid allows for the detection of VP6, i.e., DLPs, to be accessible for staining. **(B)** Graph showing a higher positive detection of anti-RV-FITC compared to anti-DLP at 0 min. **(C)** Graph showing a higher positive detection of anti-DLP compared to anti-RV-FITC at 30 min. **(D)** Relative percentage of internalised virus from untreated and inhibitor-treated cells at 30 min post-infection is calculated by dividing the relative internalised RV (30 min) **(C)** by relative attached RV (0 min) **(B)**. Error bars indicate the standard error of the mean (*n* = 3). Lowercase letters indicate significant differences (*p* < 0.05) compared to the control.

The authors apologize for this error and state that this does not change the scientific conclusions of the article in any way. The original article has been updated.

