# Supplementary figures and images for: Rotavirus-Mediated Prostaglandin E2 Production in MA104 Cells Promotes Virus Attachment and Internalisation, Resulting in an Increased Viral Load
Source: Front Physiol. 2022 Jan 28;13:805565. doi: 10.3389/fphys.2022.805565 (PMC8831913; doi:10.3389/fphys.2022.805565)

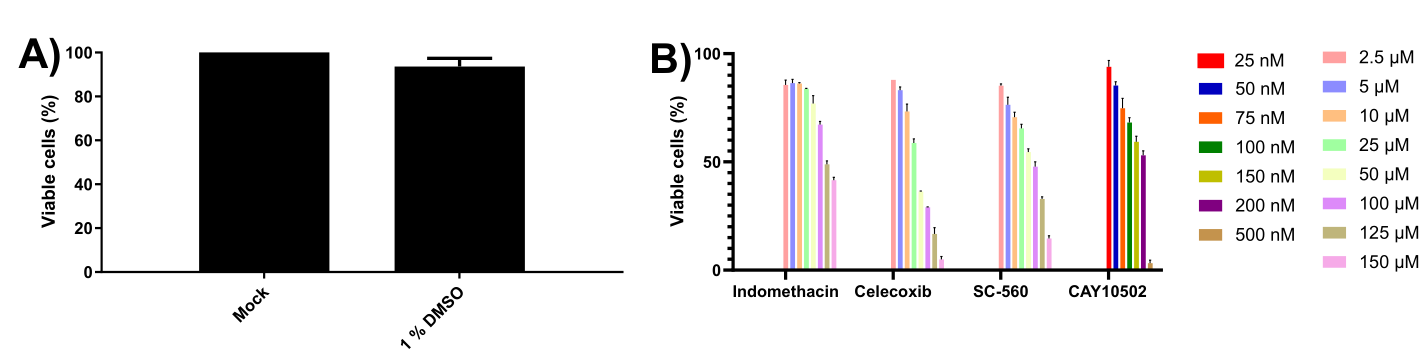

Supplement: Supplementary Figure S1 — Cellular toxicity of the inhibitors and DMSO was evaluated with the XTT assay. MA104 cells (4 × 10^4^ cells/ml) were seeded into 96-well plates and allowed to grow to 100% confluence. The inhibitors were added 1 h prior to infection and subsequently every 4 h till the completion of the experiment. (A) All inhibitors were added to MA104 cells containing a final concentration of 1% DMSO to ensure the vehicle had no detrimental effect on the cells. Results indicate that 1% decreases cell viability to approximately 92 ± 3%. (B) Data obtained from the toxicity assays. Concentrations to be used for the different inhibitors were determined as follows: indomethacin 25 μM (84% ± 0.19), celecoxib 5 μM (85% ± 0.85), SC-560 2.5 μM (83% ± 1.5) and CAY10502 25 nM (93% ± 2.8). [file Image_1.tiff]

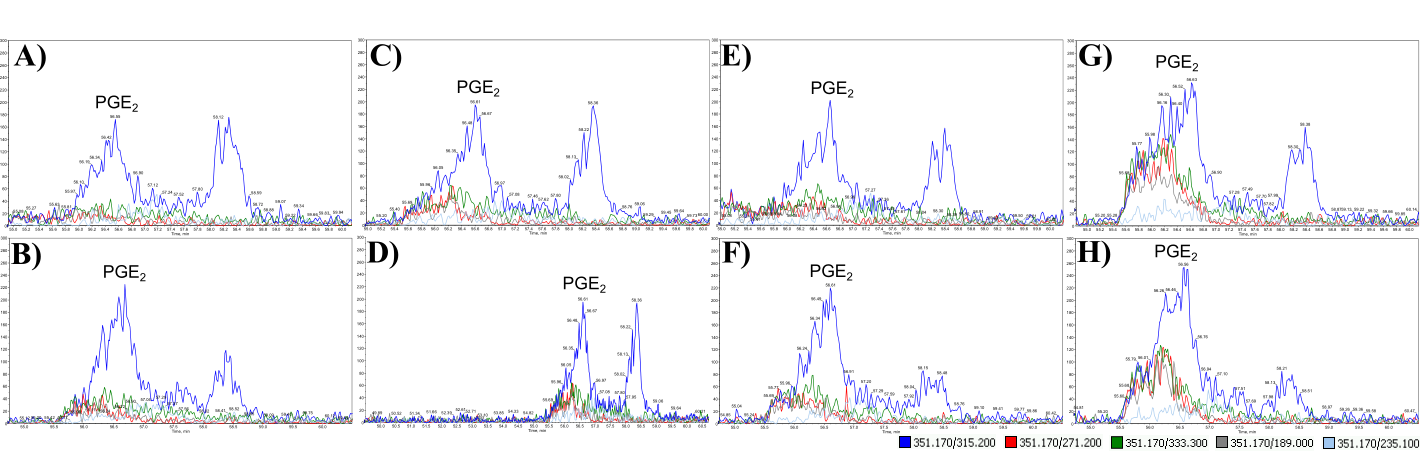

Supplement: Supplementary Figure S2 — Mass spectra showing the five transitions (Multiple reaction monitoring) for each of the samples. Only one transition of PGE2 is present in the mock infections at (A) 2 h and (B) 4 h. All five the transitions of PGE2 are present in cells infected at a MOI 0.1 (C,D) and 1 (E,F) at 2 h (C,E) and 4 h (D,F) post-infection, respectively. Furthermore, all five the transitions of PGE2 are present in cells infected at a MOI of 10 t (G) 2 h and (H) 4 h post-infection. Due to very low signal to noise ratios, the LC-MS/MS data were only used to verify the presence of PGE2. Peaks show the relevant transitions 351.17 > 315.2; 351.17 > 271.2; 351.17 > 333.3; 351.17 > 189.0; and 351.17 > 235.1. Prostaglandin E2 (PGE2). [file Image_2.tiff]
